# Exploring the Significance of Gut Microbiota in Diabetes Pathogenesis and Management—A Narrative Review

**DOI:** 10.3390/nu16121938

**Published:** 2024-06-19

**Authors:** Ewelina Młynarska, Jakub Wasiak, Agata Gajewska, Greta Steć, Joanna Jasińska, Jacek Rysz, Beata Franczyk

**Affiliations:** 1 Department of Nephrocardiology, Medical University of Lodz, ul. Zeromskiego 113, 90-549 Lodz, Poland; 2 Department of Nephrology, Hypertension and Family Medicine, Medical University of Lodz, ul. Zeromskiego 113, 90-549 Lodz, Poland

**Keywords:** diabetes, microbiota, SCFAs, gut–brain axis

## Abstract

Type 2 diabetes is a disease with significant health consequences for the individual. Currently, new mechanisms and therapeutic approaches that may affect this disease are being sought. One of them is the association of type 2 diabetes with microbiota. Through the enteric nervous system and the gut–microbiota axis, the microbiota affects the functioning of the body. It has been proven to have a real impact on influencing glucose and lipid metabolism and insulin sensitivity. With dysbiosis, there is increased bacterial translocation through the disrupted intestinal barrier and increased inflammation in the body. In diabetes, the microbiota’s composition is altered with, for example, a more abundant class of Betaproteobacteria. The consequences of these disorders are linked to mechanisms involving short-chain fatty acids, branched-chain amino acids, and bacterial lipopolysaccharide, among others. Interventions focusing on the gut microbiota are gaining traction as a promising approach to diabetes management. Studies are currently being conducted on the effects of the supply of probiotics and prebiotics, as well as fecal microbiota transplantation, on the course of diabetes. Further research will allow us to fully develop our knowledge on the subject and possibly best treat and prevent type 2 diabetes.

## 1. Introduction

Diabetes mellitus (DM) is a group of chronic metabolic conditions defined by defects in insulin production, sensitivity, or both, resulting in hyperglycemia [1]. Traditionally, it has been classified into type 1 DM, which is insulin-dependent, autoimmune-mediated, and results from the destruction of β-cells of the pancreas, and type 2 DM, which is non-insulin dependent, and is a result of insufficient insulin secretion and increased insulin resistance [2].

The chronic elevation of blood glucose levels results in microvascular changes, such as diabetic neuropathy (e.g., gastroparesis, diabetic symmetric polyneuropathy (DSPN), enteropathy, and diabetes-related hypoglycemia unawareness), eye disease, including diabetic retinopathy and cataracts, and diabetic chronic kidney disease [3]. These are then followed by macrovascular changes, such as coronary heart disease, peripheral artery disease, cerebrovascular disease, and sudden death. Undiagnosed or poorly controlled DM can result in acute, potentially life-threatening consequences, such as nonketotic hyperosmolar syndrome or hyperglycemia with ketoacidosis [4].

This multiorgan involvement in DM complications results in greater mortality of those affected [5]. Consequently, it is acknowledged as a burden on public health in association with the costs of medical healthcare, early death, and societal costs. This has led to the pursuit of new therapeutic options and a greater understating of the pathophysiology of DM. Recent advancements in treatment have introduced new pharmacotherapies, targeting key pathways such as GLP-1Ras, DPP-4, and SGLT-2 inhibitors, which influence the progression of T2DM and its complications [6,7].

Approximately 90% to 95% of DM cases are diagnosed as DMT2, which is related to multi-genetic and environmental factors [8]. Age, obesity, a sedentary lifestyle, smoking, and eating a diet high on the glycemic index, low in dietary fiber content, and rich in specific dietary fatty acids all raise the risk of developing this kind of DM [9].

It has recently been proposed that the interaction between a host’s genome and their dietary choices can cause microbiome alterations that cause DM and its consequences to develop [10]. As microbiome disruption mediates oxidative stress and chronic inflammation, it contributes to the pathophysiology not only of diabetes, but also of many more ailments, such as obesity, allergies, cancer, and cardiovascular disease [11].

## 2. The Microbiota and the Consequences of Its Disorders

**Figure 1:**
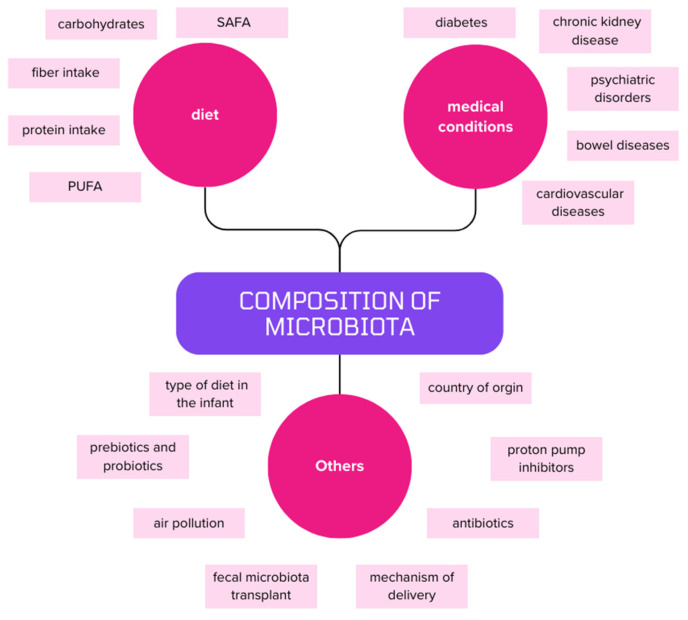
A simplified representation of the factors affecting the composition of the microbiota. PUFA—polyunsaturated fatty acid; SAFA—saturated fatty acid.

The term microbiota refers to the microbes found in the human gastrointestinal tract. Its composition is quite individual and changes during our lives, reaching a certain stability in adulthood. The beginning of its development takes place in the fetal stage. After that, the mechanism of delivery—whether natural or by cesarean section—is very influential. The infant’s diet has an impact on the microbiota’s composition as well, especially in regard to being fed natural milk or formula feeds. As presented in Figure 1, its structure is influenced by diet, environment, drugs and antibiotics used, and the presence of medical conditions [12,13,14,15,16,17].

It is now believed that the gut microbiome of a single individual contains more than 100 trillion bacteria, and their amount also depends on the section of the gastrointestinal tract, increasing from the stomach towards the colon [18]. Their genetic material contains 100 times more genes than the human genome, and their estimated mass in an organism is about 1 to 3 kg [19,20]. As mentioned, the composition of the microbiota is quite unique, but in healthy individuals the proportions are quite similar, with 60% to 80% of the total being *Firmicutes*, 20% to 40% being *Bacteroidetes*, and *Proteobacteria* and *Actinobacteria* making up about 5% [18,21,22]. It is thought that the composition of microbiota with a high degree of variety is a sign of health. However, it is also known that a high diversity has been observed in specific disease states, although more typical is the depletion of microbiota diversity [23,24,25,26,27].

Acute diet changes have been shown to be responsible for about 20% of the microbiota variability in humans; however, long-term dietary changes have a greater impact on microbiota composition [28]. Depending on the food we provide to our body, affecting the development of specific species of bacteria, we distinguish three main enterotypes of microbiotas. The first has a high percentage of *Bacteroides* and it is characteristic of the so-called Western diet in usually highly industrialized countries, where a low-fiber, high-fat diet is the norm. Enterotype 2 is dominated by *Prevotella* and is characteristic of less industrialized countries, where less processed foods and more fiber are eaten. The latter contains increased amounts of *Ruminococcus* and occurs like enterotype 1 mainly in those who eat large amounts of protein and fat [29,30].

The state of the normal coexistence of beneficial types of microbes is called eubiosis [31]. It is important for our functioning due to the existence of the microbiota–gut–brain axis, meaning bidirectional communication between the gut microbiota and the brain via the enteric nervous system (ENS), vagus nerve, immune system, neurotransmitters, short-chain fatty acids (SCFAs), and more [25,32]. An estimated 5–10% of our daily energy comes from the fermentation processes carried out by the gut bacteria [33].

Changes in gut secretion, motility, and immunological regulation can be attributed to the effects of both the autonomic nervous system through the vagus nerve and the enteric nervous system (ENS), composed of enteric neurons and enteric glial cells. In this way, our body can also independently influence the composition of the microbiota [34,35]. Conversely, gut microbes can communicate with the central nervous system (CNS) via neuronal, hormonal, and immune signaling channels and, through this, affect the entire body in several ways. Through the hyperacetylation of histones, SCFAs (acetate, propionate), produced by intestinal bacteria, can control the expression of human genes. SCFAs, which originate from the microbial breakdown of fibers, also have systemic effects, e.g., immunomodulation, appetite regulation, calcium absorption, and glucose homeostasis [36,37]. The microbiota also affects CNS function through neurotransmitters (acetylcholine, catecholamines, and gamma-aminobutyric acid) and biogenic amine (histamine) production, or changes in tryptophan metabolism [38,39].

The phrase dysbiosis refers to a disruption in the intestinal microbiota’s composition and function, and has a negative impact on the functioning of the body [40]. Firstly, this leads to a pathological condition of increased intestinal permeability due to a disruption in intestinal barrier function, which is called “leaky gut” syndrome (LGS) [17,41]. This is associated with the translocation of bacterial components, toxic metabolites, and inflammatory factors into the bloodstream, particularly exacerbated when dysbiosis occurs [42]. One such molecule is bacterial lipopolysaccharide (LPS), which in our body is recognized by Toll-like transmembrane receptors (TLRs), specifically TLR4 [43]. This leads to the production of pro-inflammatory cytokines such as tumor necrosis factor-alpha (TNF-α), IL-6, IL-8, and IL-12, followed by the appearance of both local and systemic inflammation [17,44,45].

Chronic inflammation disrupts the hypothalamic–pituitary–adrenal (HPA) axis, which is essential for the release of fuels high in energy, such glucose, amino acids, and free fatty acids, which support the immune system when it is engaged. The dysregulation of the HPA axis is followed by an increased release of glucocorticoids and catecholamines. This influences glucose metabolism by suppressing insulin secretion, promoting gluconeogenesis in the liver, suppressing glucose uptake, and inflicting insulin resistance [46,47].

Moreover, the low-grade systemic inflammation occurring in LGS leads to the impairment of the brain blood barrier (BBB), which, by controlling the flow of chemicals, ions, and cells to the brain, is essential for preserving brain homeostasis and proper neuronal activity [48,49]. Increased BBB permeability leads to a reduced number and impaired function of astrocytes, or the hyperactivity of the microglia [39,50]. It has been shown that these changes can predispose individuals to the onset of psychiatric disorders such as depression and Alzheimer’s disease [51,52].

In addition, the enlarged flow of nutrients and bacterial antigens into the bloodstream when there is increased intestinal permeability causes T-cell activation, which can lead to the development of chronic inflammatory diseases in the gut. Or, they can be carried via the blood to multiple different organs, activating the autoimmune reaction in both genetically vulnerable and non-susceptible individuals [42,45].

As mentioned, microbiota composition disorders occur in many medical conditions. As recent studies show, dysbiosis is characteristic of many gastroenterological disorders and diseases, such as gastroparesis, irritable bowel syndrome, chronic constipation, gastroesophageal reflux disease, and Barrett’s esophagus. In addition, these links are found in many other diseases, such as psychiatric disorders, hypertension, cardiovascular diseases, metabolic disorders, and more. The mechanisms underlying the above-mentioned connections are complex and are still under investigation. However, they are linked to the described increases in intestinal permeability, bacterial translocation, chronic low-grade inflammation, and substances secreted by the microbiota [14,53,54,55,56,57,58,59].

### 2.1. Compositional Changes in Microbiota of T2DM Patients

Multiple recent studies suggest that diabetes mellitus type 2 (T2DM) can be related to some compositional changes in the gut microbiota. In one of the first studies on this subject, Larsen et al. noted significantly lower levels of class Clostridia and phylum Firmicutes in the microbiota of T2DM patients than the controls [60]. Additionally, positive correlations were found between plasma glucose concentration and the ratios of Bacteroidetes to Firmicutes and Bacteroides-Prevotella to C. coccoides-E. rectale. The class Betaproteobacteria was also more prevalent in T2DM patients, which was positively associated with reduced glucose tolerance. A metagenome-wide association study (MGWAS), conducted by Qin et al. in a group of 345 Chinese individuals with or without T2DM, confirmed microbial dysbiosis in the patients suffering from T2DM [61]. The intestinal microbiota of patients with T2DM was identified by an elevated quantity of opportunistic pathogens, including Bacteroides caccae, Clostridium hathewayi, Clostridium symbiosum, Eggerthella, lenta Clostridium ramosum, and Escherichia coli, with a considerably decreased amount of various butyrate-producing bacteria, such as Clostridiales *sp.* SS3/4, E. rectale, Faecalibacterium prausnitzii, Roseburia intestinalis, and Roseburia inulinivorans, compared to the healthy controls. An increased number of the mucin-degrading species Akkermansia muciniphila, as well as the sulphate-reducing species Desulfovibrio, was also reported in the microbiome of T2DM patients [61]. Karlsson et al., in their study on a European population of women with T2DM, confirmed the decrease in Roseburia intestinalis and Faecalibacterium prausnitzii species in patients with T2DM [62]. Furthermore, a higher abundance of four Lactobacillus species and decreased levels of five Clostridium species were found in patients suffering from T2DM, compared to the normal glucose tolerance patients [62]. Significantly, levels of Lactobacillus had positive correlations with fasting glucose and glycosylated hemoglobin (HbA1c), while Clostridium was negatively associated with fasting glucose, HbA1c, plasma triglycerides, C-peptide, and insulin, suggesting that the above-mentioned species could be linked to the development of T2DM. Interestingly, some authors suggest that shifts in the composition of the gut microbiome in T2DM patients may be partially influenced by metformin, a widely prescribed antidiabetic medication that is characterized by a notable rise in abundance of Escherichia species and a decrease in taxa associated with butyrate production [63].

### 2.2. The Role of the Gut Microbiota in the Pathogenesis of T2DM

**Figure 2:**
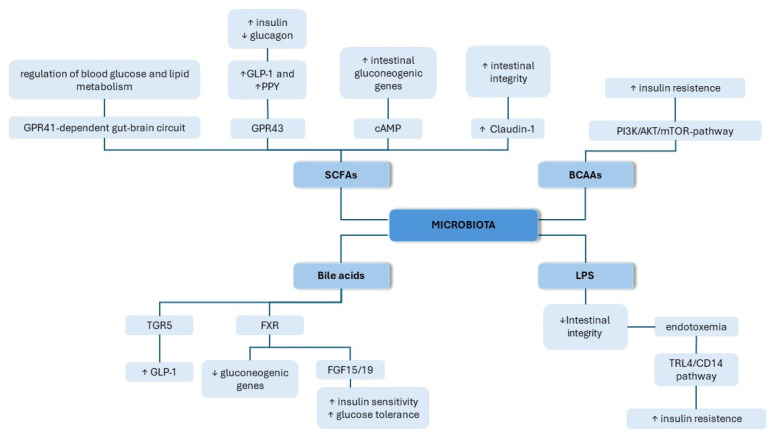
The main mechanisms of gut microbiota involvement in the development of T2DM. BCAAs—branched-chain amino acids; FGF15/19—fibroblast growth factor 15/19; FXR—farnesoid X receptor; GLP1—glucagon-like peptide-1; PPY—peptide YY; SCFAs—short-chain fatty acids; TGR5—Takeda G protein-coupled receptor; LPS—lipopolysaccharide; TRL4—Toll-like receptor 4.

Dysbiosis contributes to the pathogenesis of T2DM through various molecular mechanisms, as shown in Figure 2. The gut microbiota can affect the development of diabetes by influencing glucose and lipid metabolism, insulin sensitivity, and inflammation, as well as affecting gut permeability. The effects mentioned above can be evoked through various signaling pathways with the involvement of important regulators, such as SCFAs, bile acids, branched-chain amino acids (BCAAs), and LPSs. In this section, we provide an overview of the different modes of action underlying the contribution of the microbiota to the course of diabetes.

#### 2.2.1. SCFAs

SCFAs are metabolites produced by intestinal microbiota in the process of dietary fiber fermentation [64]. The three main SCFAs are butyrate, propionate, and acetate, and in many studies, they are suggested to have an impact on glucose metabolism and insulin sensitivity, thereby affecting the development of diabetes. Using bidirectional Mendelian randomization (MR) analyses to assess causality, Sanna et al. observed that the intensification in intestinal production of the SCFA butyrate, driven by host genetics, was affiliated with better insulin response following an oral glucose-tolerance test [65]. Butyrate has also been demonstrated to promote the release of glucagon-like peptide-1 (GLP-1) and peptide YY (PPY) via L cells in the colon, two gut-secreted hormones that are responsible for delaying gastric emptying, reducing appetite, facilitating insulin secretion, and suppressing glucagon [66]. Moreover, butyrate is capable of influencing glucose homeostasis by increasing intestinal gluconeogenesis [67]. Through a cAMP-dependent mechanism, butyrate stimulates the expression of intestinal genes responsible for gluconeogenesis in the gut [67]. Butyrate also improves the integrity of the intestinal barrier by enhancing the transcription of Claudin-1 via promoting the interaction between transcription factor SP1 and the Claudin-1 promoter region, leading to a redistribution of ZO-1 and Occludin on the cell membrane [68]. Additionally, Sanna et al. proved a causal relationship between disruptions in the production or absorption of another SCFA, propionate, with a heightened risk of T2DM [65]. In another study, propionate was demonstrated to stimulate the release of GLP-1 and PPY in rats and mice, acting through the GPR43 receptor [69]. Moreover, propionate promotes also intestinal gluconeogenesis by affecting the GPR41-dependent gut–brain neural circuit, thereby regulating blood glucose and lipid metabolism [67]. In a randomized control trial, the acute supplementation of propionate increased PYY and GLP-1 secretion in humans, as well as reduced food intake [70]. In long-term supplementation, propionate caused weight gain reduction and prevented the deterioration of insulin sensitivity in overweight adults [70].

#### 2.2.2. BCAAs

Branched-chain amino acids (BCAAs) are essential amino acids, meaning they cannot be synthesized by the host. Their source in the human body mostly originates from the diet and microbial production [71]. Growing evidence suggests the link between the elevated serum levels of BCAAs and the occurrence of diabetes or insulin resistance [72,73]. The increased dietary intake of BCAAs has been proven to promote the progression of insulin resistance and T2DM [74,75]. BCAAs have been found to promote insulin resistance through the mTOR signaling pathway. Mice fed with BCAAs exhibited elevated expressions of phosphorylated mTORSer2448, phosphorylated S6K1Thr389, and phosphorylated IRS1- Ser302, which can induce changes in insulin signaling, resulting in insulin resistance [76]. In another study, BCAA intake significantly elevated hepatic gluconeogenesis and inhibited hepatic adipogenesis in high-fat-diet-fed mice by inhibiting Akt2 signaling via mTORC1- and mTORC2-dependent pathways [77]. BCAAs are also capable of activating phosphatidylinositol 3 kinase (PI3K), which induces AKT phosphorylation, leading to higher insulin resistance [78]. However, additional research needs to be performed to enhance the comprehension of the exact molecular mechanism underlying this process.

#### 2.2.3. Bile Acids

Primary bile acids are synthetized in the liver as a product of cholesterol metabolism. They are further released into the intestine and converted by colonic microbiota into secondary bile acids [79,80]. High serum levels of bile acids have positive effects on glucose metabolism, improve insulin sensitivity, and are linked to better postprandial blood sugar regulation [81,82]. Secondary bile acids have been reported to stimulate the secretion of GLP-1 from intestinal L cells by activating the Takeda G protein-coupled receptor (TGR5) [83,84,85]. In obese and insulin-resistant murine models, Thomas at al. proved that mice with a gain of function of TGR5 show better glucose tolerance, while in TGR5^−/−^ mice, glucose tolerance is impaired [86]. The effect was linked to a healthier pancreatic islet phenotype and could be partially explained by the insulinotropic effect following TGR5 activation. Bile acids can also influence glucose metabolism by activating the nuclear farnesoid X receptor (FXR) [87]. In in vitro studies, the bile-acid-related activation of FXR suppressed the expression of gluconeogenic genes, such as glucose-6-phosphatase, phosphoenolpyruvate carboxykinase, and fructose-1,6-bisphosphatase [88]. Additionally, FXR activation also results in the release of fibroblast growth factor 15/19 (FGF15/19), which exerts beneficial effects on glucose tolerance and insulin sensitivity [89,90]. FXR was also found to positively influence glucose tolerance and weight loss maintenance following sleeve gastrectomy, and the effect correlated with the increase in the serum concentrations of bile acids, as well as changes in the composition of the intestinal microbiota [91]. These data indicate that bile acids play a significant role in the pathogenesis of T2DM by influencing various signaling pathways.

#### 2.2.4. LPS

LPS is a constituent of Gram-negative bacteria’s outer walls that has emerged as a potential contributor to the pathogenesis of T2DM. It contributes to intestinal permeability, which has been associated with gut microbiome disruption [92]. Diets with a high fat content are linked to a larger proportion of LPS-containing microbiota, and thus a high plasma LPS concentration. High-fat diets have been shown to elevate plasma LPS levels through increased intestinal permeability, or through LPS uptake in chylomicrons, which are secreted from intestinal epithelial cells [93,94]. Increased LPS levels can potentially disrupt the integrity of the intestinal barrier and result in further LPS absorbtion [95]. LPS is an effective activator of the TLR4/CD14 pathway, through which it triggers an inflammatory response that has been suggested to add to systemic insulin resistance [96]. The activation of TLRs results in the translocation of NF-κB and initiates the transcription of IL-6, IL-1, and TNF-α [97]. Lin et al. found that 2h post the administration of LPS, diabetic mice had four times greater concentrations of peritoneal IL-1β, IL-6, and TNF-α [98].

Through the activation of the TLR4 pathway and the release of IL-6, LPS contributes to insulin secretion. Pancreatic β-cells increase the production and secretion of insulin as a result of a greater reaction to glucose through the Il-6 stimulation of glucagon-like peptide 1 (GLP-1) in L cells [99].

Furthermore, an upregulation of soluble CD14, a monocytic marker for LPS activity, has previously been reported in T2DM subjects [100]. Using CD14 mutant mice, Cini et al. demonstrated a causal link between T2DM and LPS. In wild-type mice, it was found that a 3 h intravenous LPS infusion significantly increased Il-6, PAI-1, and IL-1 mRNA concentrations in subcutaneous adipose tissue with a markedly dull increase in CD14 mutant mice. LPS infusion induced 4-week endotoxemia and caused an elevation in fasted glycemia, insulinemia, and whole-body weight gain comparable to mice fed a high-fat diet. CD14 mutant mice avoided the majority of features of metabolic disease stimulated by a high-fat diet or LPS infusion [101].

## 3. Gut Microbiota—Potential Therapeutic Target for the Management of Diabetes

Recent studies have demonstrated the complex relationship between the composition of the gut microbiota and the metabolism, with alterations in microbial communities related to the beginning and further development of diabetes. Interventions that focus on the gut microbiota are gaining attention as a promising approach for diabetes management, with several strategies being investigated to positively influence the gut microbial community and thereby improve metabolic health [102].

Probiotics have received attention for their possible role in improving glycemic control and insulin sensitivity, particularly in individuals with diabetes or those at risk of developing the disease. Probiotics are live microorganisms that, when properly used, give health advantages to the host, mostly by improving or restoring the gut microbiota [102,103]. Several clinical investigations have shown that particular probiotic strains enhance glucose management. The most frequently studied bacterial strains in the trials, Lactobacillus acidophilus, Bifidobacterium bifidum, and Lactobacillus casei, were found to have beneficial effects on a variety of metabolic processes [104].

Probiotics and their metabolites exert a substantial impact on insulin resistance and metabolic health in T2DM. Certain probiotic bacteria, such as Lactobacillus and Bifidobacterium, may produce SCFAs during the fermentation of dietary fibers in the intestines, which has been related to a variety of health benefits in the context of T2DM. A major mechanism includes the interaction of SCFAs with G-protein-coupled receptors (GPCRs), particularly GPR41 and GPR43. GPR43-activated SCFAs can enhance insulin sensitivity and alleviate inflammation by improving glucose absorbtion and triggering glucagon-like peptide-1 (GLP-1) release, which stimulates insulin secretion [105,106,107]. SCFAs also participate in interactions with nuclear receptors, such as peroxisome proliferator-activated receptors (PPARγ). PPARγ activation promotes adipocyte differentiation and decreases inflammation, improving insulin sensitivity [108,109]. Probiotics can also modulate immunological activity and reduce the pro-inflammatory cytokines levels linked to insulin resistance by interacting with Toll-like receptors (TLRs). For instance, L. plantarum C88 attenuates the inflammatory reaction by notably lowering the blood concentrations of pro-inflammatory factors, including TNF-α, IL-1β, IL-6, IL-8, and IFN-γ. Moreover, L. plantarum C88 decreases the nuclear factor kappa B (NF-κB) signaling pathways by reducing the activation of TLR2 and TLR4, and inhibits NF-κB nuclear translocation by increasing the synthesis of its antagonist [110].

One study, conducted by Khalili et al., investigated the consequences of L. casei supplementation on the blood sugar levels and Sirtuin 1 (SIRT1) and fetuin-A levels of patients with type 2 diabetes [111]. In diabetes, SIRT1 enhances insulin sensitivity by deacetylating insulin receptor substrate 2 (IRS2), which boosts the insulin regulatory pathway. SIRT1 also supports mitochondrial biogenesis and reduces gluconeogenesis, aiding in better glucose metabolism. Disruptions in SIRT1 activity are linked to T2DM and other metabolic dysfunctions; decreased SIRT1 expression and activity have been linked to poor insulin signaling and glucose intolerance [112,113]. Therapeutic strategies that target SIRT1 activity have shown promise in increasing insulin sensitivity and glycemic control in diabetes [114]. Fetuin-A is a liver-derived protein that can suppress insulin signaling by inhibiting insulin receptor autophosphorylation. Higher levels of fetuin-A are linked to insulin resistance, T2DM, and increased inflammation. It also promotes ectopic lipid accumulation, exacerbating metabolic dysfunction and contributing to insulin resistance and insufficient glycemic management in T2DM [115]. The findings of this randomized controlled trial demonstrated that, in comparison with the placebo population, participants who took L. casei supplementation for eight weeks exhibited enhancements in their preprandial blood glucose concentration, insulin concentration, and insulin resistance. Furthermore, following treatment, HbA1c decreased, although the decrease was not clinically significant. By the end of the trial, the L. casei supplementation also significantly reduced fetuin-A levels and raised SIRT1 when compared to the placebo. These results imply that L. casei supplementation may be advantageous for improving glycemic control in individuals with diabetes [111].

Beyond its impact on glucose regulation, Lactobacillus casei may also possess anti-inflammatory properties, potentially extending its potential benefits for diabetes management. Insulin resistance and T2DM are correlated with sustained low-grade inflammation, and probiotics such as Lactobacillus casei have been demonstrated to lower inflammation indicators, including CRP and TNF-α [116,117].

Prebiotics, like fructooligosaccharides, galactooligosaccharides, and inulin, are indigestible dietary fibers or substances that stimulate the formation and functioning of beneficial bacteria in the digestive system and may influence general health [118]. Prebiotics were shown to significantly reduce HbA1c in comparison to the control group, with a standardized mean difference [119]. A meta-analysis found that inulin supplementation significantly improved preprandial blood glucose, the Homeostasis Model Assessment of Insulin Resistance (HOMA-IR), and HbA1c. Subgroup analyses indicated that inulin was most effective when treatment lasted at least eight weeks. These results suggest that inulin could be beneficial in managing type 2 diabetes, especially with longer treatment durations [120].

Synbiotics, which combine probiotics and prebiotics, also offer a promising approach to managing diabetes by leveraging the combined benefits of both to improve gut health and glycemic control. A randomized, double-blind, placebo-controlled clinical trial of 60 individuals with diabetes undergoing hemodialysis is one example of a study evaluating the impact of synbiotics on DM2. Patients were randomized to either a synbiotic group, receiving Lactobacillus acidophilus, Lactobacillus casei, Bifidobacterium bifidum, and inulin, or a placebo group, both for a duration of 12 weeks. The results showed that synbiotic supplementation significantly lowered fasting blood glucose, insulin concentration, and insulin resistance, while enhancing insulin sensitivity. Furthermore, the synbiotic group experienced notable reductions in high-sensitivity CRP and malondialdehyde, indicators of inflammation and oxidative stress, respectively. Additionally, the glutathione levels and total antioxidant capacity were higher in the same group, suggesting a boost in the body’s antioxidant defenses [121].

**Figure 3:**
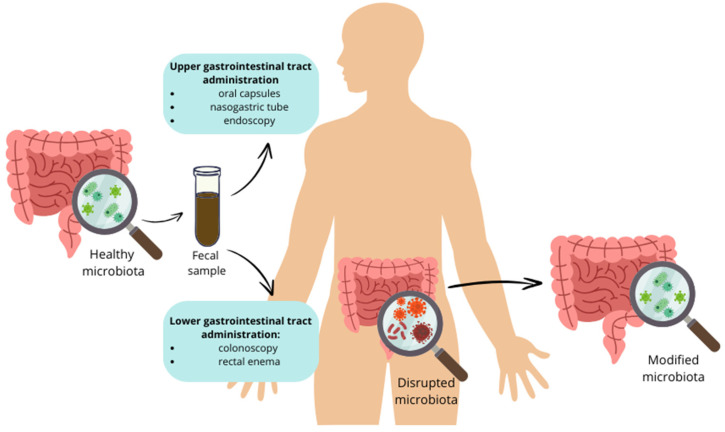
A simplified scheme of fecal microbiota transplantation.

Fecal microbiota transplantation (FMT) is a therapeutic procedure that involves transplanting fecal bacteria and other microbes from one healthy individual to another individual. A schematic image of the FMT process is shown in Figure 3. Although FMT is used primarily to deal with recurring infections caused by Clostridium difficile, it is also garnering attention as a possible therapy for metabolic disorders such as T2DM. Thirty-one individuals with recently diagnosed T2DM were randomly assigned into three groups—metformin, FMT, and FMT plus metformin—and followed for 4 weeks. Wu et al. evaluated the effects of FMT, metformin, and their combination on insulin resistance and gastrointestinal microbiota in T2DM. Both FMT independently and FMT combined with metformin notably enhanced key clinical indicators in patients with T2DM. Improvements were observed in the HOMA-IR and Body Mass Index (BMI), along with reductions in both preprandial and postprandial blood glucose, and HbA1c levels. FMT treatments increased the gut microbial diversity in T2DM patients through the successful colonization of donor microbiota. The group that received both FMT and metformin experienced a more significant shift in their gut microbiota, with 441 microbial species altered compared to 227 species in the FMT-only group. This suggests that the combined approach leads to a greater impact on the gut microbiota. These results suggest that FMT, whether used independently or in combination with metformin, can positively impact several critical measures of diabetes control [59].

Finally, diet and exercise also play crucial roles in modulating the gut microbiota, with significant implications for diabetes management. High-fiber diets high in fruits, vegetables, and whole grains have been shown to sustain a diverse and beneficial microbial environment, increasing the production of SCFAs, which improve insulin sensitivity and reduce inflammation [122]. Regular physical activity also impacts the gastrointestinal microbiota. Studies have demonstrated that exercising can enhance the inflammatory state, blood glucose regulation, and insulin sensitivity in individuals with T2DM. Animal studies also confirm the positive influence of exercise training on the gut microbiota. According to Evans et al., in mice given a high-fat diet, physical training restored a disturbed gut microbiota, as shown by a negative association between exercising and the Bacteroidetes/Firmicutes ratio. A different study revealed that exercising mice demonstrated a greater concentration of SCFAs and an altered gut microbiota composition compared to non-exercising mice. These results suggest that exercise can help restore gut health and increase SCFA production [123]. Combining a diet with regular exercise creates a holistic approach that promotes a healthier gut microbiota, increases insulin sensitivity, and reduces inflammation, helping in diabetes management.Interventions targeting the gut microbiota show promise for improving metabolic health and managing diabetes. Ongoing research, including clinical trials, is being undertaken to study the impact of gut microbiota interventions on T2DM control to identify the most effective approaches. The Table 1 summarizes the microbiota-targeted interventions and their impact on diabetic patients.

Individual variability is an important consideration: the goal should be establishing individual strategies that consider an individual’s unique profile, resulting in more successful and long-term diabetes control. Additional research is needed to identify the best dosages, strains, and treatment durations for these treatments, as well as their long-term safety and efficacy. Expanding our knowledge of the gut microbiota’s function in diabetes allows us to develop comprehensive treatment programs that integrate these microbial-based methods with other recognized medications, ultimately providing the best diabetes management.

## 4. Gut Microbiota—Future Directions

As our understanding of the gut microbiome’s influence on diabetes continues to evolve, there is growing recognition of the potential for microbiota-targeted strategies to revolutionize diabetes management.

One possible future avenue is the development of individualized microbiome-based therapeutics suited to individual gut microbiota structures. This method might help with the design of tailored therapies, such as probiotic or prebiotic formulations, dietary recommendations, or FMT, personalized to each patient’s specific microbiome composition [129].

In addition to customized interventions, future research may concentrate on the development of microbiota-targeted pharmacotherapies. Novel medical therapies that particularly target the gut microbiota could be investigated to influence microbial composition and activity, thereby improving metabolic outcomes in diabetic patients. These therapies may include microbial metabolite modulators, microbial enzyme inhibitors, or microbiota-targeted antibiotics. Microbial metabolite modulators may affect the production and activity of certain microbial metabolites involved in metabolic dysfunction, such as SCFAs or bile acids. Similarly, microbial enzyme inhibitors may target critical microbial enzymes implicated in metabolic pathways that contribute to diabetes development. Microbiota-targeted antibiotics selectively identify and remove pathogenic or dysbiotic bacteria species involved in diabetes etiology. Such therapies have the potential to restore microbial balance and promote metabolic health by reducing the quantity of harmful bacteria while increasing the growth of beneficial ones [130].

Microbiota-targeted vaccines represent a promising avenue for future interventions in diabetes management. These vaccinations might function by activating the immune system to develop specific responses to harmful bacteria while protecting beneficial ones. Furthermore, they may reduce inflammation and enhance immunological tolerance, resulting in a reduction in the chronic low-grade inflammation associated with diabetes and related complications [131].

Investigating microbiota-based gene treatments is a revolutionary approach to diabetes management. These therapies may involve targeting host or microbial genes implicated in diabetes pathogenesis to modulate gene expression. Targeting the specific genes involved in metabolic pathways, including glucose metabolism, insulin signaling, and inflammation, may correct the underlying metabolic abnormalities associated with diabetes [132].

Finally, additional modifications to dietary therapies focused on altering gut microbiota composition and function could be investigated. This could include developing personalized nutritional plans based on individual microbiome profiles, as well as identifying specific food components or bioactive chemicals that modulate the microbiota [133].

These novel treatment techniques open up new possibilities for future study and development in the field of microbiota-targeted therapy for diabetes management. These methods have the potential to revolutionize diabetes therapy and improve patient outcomes by embracing the complicated interactions between the gut microbiota and the host metabolism.

## 5. Conclusions

In conclusion, T2DM is a metabolic disease with prevalence rising globally. T2DM is a leading cause of blindness, stroke, lower limb amputation, and kidney failure. The recognition of T2DM as a health and social burden has led to extensive research to alleviate the costs of this disease by broadening our understanding of the factors involved in its pathogenesis and progression, and finding new therapeutic options. A progressed understanding of the microbiome and its interaction with metabolic health has led to research concerning its influence on T2DM.

The intricate interaction between the gut microbiota and T2DM highlights the multicausal pathophysiology of this and other metabolic diseases. Through a comprehensive review and analysis, this article has explained the mechanism of microbiome dysbiosis as a possible factor in the pathogenesis of T2DM, and potential treatment options for microbiota-targeted interventions. The composition shifts in the microbiome of T2DM-affected individuals are embodied by a reduction in butyrate-producing bacteria and a rise in opportunistic pathogens. Furthermore, dysbiosis-related changes in SCFAs, BCAAs, and LPSs have been observed to contribute to chronic inflammation, insulin resistance, and impaired glucose metabolism. With the rapid development of new treatment options for T2DM, microbiota-targeted interventions offer a promising new path of management. The administration of probiotics, prebiotics, symbiotics, and FMT has been proven effective in improving sensitivity to insulin and optimizing blood glucose level management in patients with T2DM. Lactobacillus and Bifidobacterium strains have shown promising effects in modifying gut microbiota composition and metabolic outcomes through immune modulation, gut barrier integrity maintenance, and SCFA production.

Additionally, lifestyle interventions including increased physical activity and nutritional changes play an integral role in microbiome modifications and the improvement of metabolic health in those with T2DM. A diet high in fiber, whole grain produce, fruits, and vegetables promotes a diverse microbial community, which is beneficial for increasing the production of SCFAs, leading to improved insulin sensitivity. Subsequently, research has demonstrated an inverse relationship between exercise and Bacteroides/Firmicutes ratio, further proving that exercising regularly does have a favorable effect on gut microbiota and its metabolism. Nonetheless, despite the evidence supporting the effectiveness of T2DM interventions targeted towards microbiota modulation, several challenges persist. Firstly, additional investigation is necessary to confirm the long-term safety of such treatments. The administration of probiotics can lead to three theoretical concerns. One of which is the occurrence of diseases such as endocarditis or bacteriemia, followed by toxic consequences for the gastrointestinal tract, and finally the transfer of antibiotic resistance to the gut microflora [134]. The safety of the administration of probiotics remains unclear. As studies have shown that probiotics may cause fungemia and bacteriemia, extra precautions should be taken when administering them to elderly or immune-compromised individuals [135,136]. This puts a potential limitation on this type of intervention in terms of controlling blood glucose levels and insulin response in wider groups of patients, as T2DM prevalence increases with age. The potential concern of intensive probiotic treatment is harboring antibiotic-resistant genes via probiotic strands and potentially transferring them to the bacterial components of the microbiome, along with potential pathogens [137]. Studies on the potential threats of long-term probiotic therapy in mice found pro-inflammatory responses and changes in the microbiome in favor of bacterial families, which promotes inflammation in the gastrointestinal tract [138].

Furthermore, as personal management plans and treatment plans are crucial for the optimization of therapeutic outcomes, future research should determine the selection of bacterial strains, dosage, means, and time of administration based on the subject’s individual microbiome composition and metabolism. Further inquiries should aim to define therapeutic goals and try to overcome the pitfalls of current T2DM management strategies. Upcoming research should focus on the potential interactions of microbiome modification and current therapeutic interventions, including drug interactions. Moreover, future investigations should aim to resolve potential adverse side-effects that have been associated with probiotics, such as bloating, diarrhea, and abdominal pain, typically occurring during the beginning of treatment or at high dosages [139]. Nevertheless, despite the potential of microbiome modification interventions, there should be an emphasis on promoting lifestyle and dietary changes, which can be undertaken separately or alongside said interventions to improve the composition and metabolism of the microbiota for a more comprehensive approach.

Lastly, the gut microbiome presents a new promising treatment objective for the control and treatment of T2DM. Continued research is crucial to further our insight into the complex relationship between the gut microbiota and metabolic health. These persistent efforts will lay the foundations for innovative and personalized treatment options for T2DM prevention and treatment. Through broadening our understanding of the therapeutic potential of the microbiota, we can develop more effective strategies to address T2DM as a global health burden and improve the quality of life of those affected.

Continued research towards an enhanced comprehension of the contribution of microbiome disruptions and its metabolism to the pathogenesis and progression of T2DM will create a new opportunity for personalized medical interventions not only in the management of T2DM, but also in other conditions.

## Figures and Tables

**Table 1:** This table depicts microbiota-targeted interventions and their impact on gut microbiota and diabetic patients.

Therapeutic Intervention	Mechanism of Action	Diabetic Patient Outcomes	References
Probiotics	Introduction of beneficial bacteria to the gut.	Improvement in glycemic control; decrease in fasting blood glucose, HbA1c, and HOMA-IR.	[124]
		Improvement in glycemic control; decrease in fasting blood glucose, HbA1c, HOMA-IR, TNF-alfa, and CRP.	[125]
Prebiotics	Non-digestible food ingredients that stimulate the growth of beneficial bacteria.	Decrease in the levels of fasting plasma glucose, HbA1c, Il-6, CRP, and TNF-alfa.	[126]
Synbiotics	Combination of probiotics and prebiotics.	Decrease in the levels of fasting plasma glucose, HbA1c, and CRP.	[127]
Dietary modifications (e.g., high-fiber diets)	Modifications in dietary habits; consuming beneficial food.	Decrease in the levels of fasting plasma glucose, HbA1c, and HOMA-IR.	[128]
Fecal microbiota transplantation (FMT)	Transplantation of stool from a healthy donor to the patient’s gut.	Decrease in the levels of fasting and postprandial plasma glucose, HbA1c, HOMA-IR, and BMI.	[59]

## Data Availability

The data presented in this study are available upon request from the corresponding author due to the partial obtainment of articles within time limits. For full-version access, contact the corresponding author.
